# Staff’s views on delivering patient-led therapy during inpatient stroke rehabilitation: a focus group study with lessons for trial fidelity

**DOI:** 10.1186/s13063-015-0646-9

**Published:** 2015-04-07

**Authors:** Maria Horne, Nessa Thomas, Andy Vail, Rudd Selles, Candy McCabe, Sarah Tyson

**Affiliations:** School of Health, University of Bradford, Horton A Building, Richmond Rd, Bradford, BD7 1DP England; Stroke Research Centre, Manchester Academic Health Sciences Centre, University of Manchester, Jean McFarlane Building, Oxford Rd, Manchester, M13 9PL England; School of Nursing, Midwifery & Social Work, University of Manchester, Jean McFarlane Building, Oxford Rd, Manchester, M13 9PL England; Salford Royal NHS Foundation Trust, Stott Lane, Eccles, Salford, M6 8HD England; Department of Plastic, Reconstructive and Hand Surgery, Erasmus MC, Postbus 2040, 3000 CA Rotterdam, the Netherlands; Department of Rehabilitation Medicine & Physical Therapy, Erasmus MC, Postbus 2040, 3000 CA Rotterdam, the Netherlands; Royal National Hospital for Rheumatic Diseases NHS Foundation Trust, Upper Borough Walls, Bath, BA1 1RL England; University of the West England, Glenside Campus, Blackberry Hill, Stapleton, Bristol, BS16 1DD England

**Keywords:** Stroke, Rehabilitation, Trial, Recruitment

## Abstract

**Background:**

Fidelity to the treatment protocol is key to successful trials but often problematic. This article reports the staff’s views on delivering a complex rehabilitation intervention: patient-led therapy during inpatient stroke care.

**Methods:**

An exploratory qualitative study using focus groups with staff involved in a multicenter (n = 12) feasibility trial of patient-led therapy (the MAESTRO trial) was undertaken as part of the evaluation process. Purposive sampling ensured that participants represented all recruiting sites, relevant professions and levels of seniority. Data analysis used a Framework Approach.

**Results:**

Five focus groups were held involving 30 participants. Five main themes emerged: the effect of the interventions, practical problems, patient-related factors, professional dilemmas, and skills. Staff felt the main effect of the therapies was on patients’ autonomy and occupation; the main practical problems were the patients’ difficulties in achieving the correct position and a lack of space. Staff clearly identified characteristics that made patient-led therapy unsuitable for some patients. Most staff experienced dilemmas over how to prioritize the trial interventions compared to their usual therapy and other clinical demands. Staff also lacked confidence about how to deliver the interventions, particularly when adapting the interventions to individual needs. For each barrier to implementation, possible solutions were identified. Of these, involving other people and establishing a routine were the most common.

**Conclusions:**

Delivering rehabilitation interventions within a trial is complex. Staff require time and support to develop the skills, strategies and confidence to identify suitable patients, deliver new treatments, adapt the new treatments to individuals’ needs and balance the demands of delivering the trial intervention according to the treatment protocol with other clinical and professional priorities.

**Trial registration:**

ISRCTN: ISRCTN29533052. October 2011.

## Background

An understanding of trial fidelity (the thoroughness to which a trial treatment protocol is adhered) is an essential element of the evaluation of complex interventions and has several benefits. Fidelity affects the statistical power of a trial because poor fidelity increases variability of the treatment delivered and thus the accuracy of any conclusions regarding efficacy. Fidelity can also provide insight into factors contributing to the results, for example, by illuminating the adherence, essential features of the intervention, or mechanisms contributing to any effect [1]. These benefits can facilitate accurate replication and implementation of procedures into everyday practice. Despite these widely acknowledged benefits, fidelity is often under-reported, especially in rehabilitation trials. Much of the literature regarding fidelity concerns its evaluation (see [2-5] for example). If addressed (sometimes as part of a process evaluation), evaluation of fidelity (often defined in terms of feasibility and acceptability) frequently relies on audits of clinical records, objective counts of recruitment and retention rates, or the amount (dose or frequency) of treatment provided, which is often complicated by issues of participant adherence [6-9]. The important issue of the professionals’ experiences while delivering the intervention within a trial is rarely considered, yet this can explain why and how treatments are delivered in the ‘real world’ and why treatments may, or may not, be adhered to.

Here we report healthcare professionals’ experience of delivering a complex intervention during a feasibility trial. Specifically, we sought to identify barriers to implementing the intervention, so strategies to overcome these problems could be developed for a definitive Phase III trial and/or eventual implementation into clinical practice. The study was part of a Phase II randomized controlled feasibility trial of usual care plus patient-led therapy during inpatient stroke rehabilitation (called MAESTRO, Mirror arm exercises after stroke; ISRCTN29533052). Two types of patient-led therapy were evaluated: mirror therapy for the upper limb and exercises for the lower limb. The groups acted as controls for each other, as the effects of therapy are specific to the targeted body areas. Thus, upper limb impairments and activity limitations were assessed for those receiving mirror therapy (with the lower limb exercise groups’ data as the control) while lower limb impairments and balance and mobility were assessed for the lower limb group (and these measures were used for control data from the mirror therapy group). The acceptability and feasibility of the interventions and the way they were delivered have been reported elsewhere [10]. The study of the staff’s experience of delivering the interventions was undertaken toward the end of the trial once the ‘treatment phase’ of the trial was complete.

Patient-led therapy involves teaching patients how to continue therapeutic exercises and activities outside formal treatment sessions. It is widely used in community rehabilitation, but is uncommon during inpatient rehabilitation. The main aim of the MAESTRO trial was to investigate the feasibility and acceptability of introducing this way of working into inpatient stroke care. This is because many stroke patients spend most of their time inactive and receive low levels of therapy [11-14], which are insufficient to develop motor skills [15]. As major increases in staffing levels are unlikely, there is an imperative to develop ways in which patients can undertake more practice and exercise without increasing the staff workload. Patient-led therapy is a possible way to achieve this.

For each participant, an individualized exercise program was devised collaboratively by the research team and treating clinician by selecting pre-prepared exercise instructions appropriate for the patient’s level of ability. The research therapist and treating clinician then taught the patient how to perform these and how to use the mirror (if randomized to the mirror therapy), provided material to remind the patients how to do the therapy, and discussed strategies for adherence (such as setting a specific time to do the therapy each day). The patient was then asked to do the patient-led therapy for up to 30 minutes daily for four weeks with ‘light touch supervision’ from a member of staff. The research therapists visited several times per week to provide advice, ongoing support and education. Before the start of the trial at each site, the researchers met with the stroke therapy team to discuss current therapy practice, the trial, the interventions and how they would be delivered at each site. Some units assigned one or two members of staff (often a therapy assistant) to be responsible for the patient-led therapy for all patients, while in other units, qualified (physical or occupational) therapists provided the light touch supervision for their patients. After therapy had been delivered for 6 months at a center,, feedback meetings on the experience were held at the center. Following this, a resource file/manual was developed and made available to all involved in the trial. This manual contained instructions on how to perform patient-led therapy, use the mirrors, and progress the exercises, plus copies of all the exercises, solutions to problems employed by different sites, and anecdotes of staff and patients’ experiences.

## Methods

To gain an in-depth understanding of the staff’s experience of implementing patient-led therapy during inpatient stroke care, we undertook focus groups. Stroke unit staff in the participating sites of the main MAESTRO trial, and who had been involved in delivering or supervising the patient-led therapies, were invited to participate in a focus group held at different locations and times of the day to facilitate attendance. Purposive sampling was used to ensure that participants represented all the recruiting sites, all relevant professions, a range of staff grades, and a range of experiences of delivering patient-led therapy. The sample size was driven by the emerging themes and determined by theoretical saturation [16]. The focus groups were led by MH, who was independent of the main trial, supported by NT. A discussion guide was developed by the authors, so the focus groups were conducted in a consistent manner [17]. Typical key questions asked about participants’ experiences of implementing the intervention, any difficulties they encountered and the strategies used to overcome them. At the beginning of each focus group, all participants gave informed consent and the facilitator clarified the aims and asked general and then more focused questions. Techniques such as clarifying, paraphrasing, open-ended and discussion questions were used to include all group members [17]. Summaries were provided frequently, and agreement and dissent were acknowledged. Each group lasted 45 to 60 minutes and was held in working hours in a hospital location convenient to the participants. Ethical approval was granted by the University and the Lancaster Committee of the National Research Ethics Service (NRES ethics reference no. 10/H1015/85)

Discussions were digitally audio-recorded, transcribed verbatim and anonymized. Data analysis and classification followed a Framework Approach [18] using NVIVO9 qualitative analysis software program for data coding, cross-referencing, storage and retrieval. There are five key stages to the analysis: familiarization; identification of the thematic framework; indexing; charting; mapping and interpretation. During the familiarization, the recordings and transcripts were listened to and read several times by the main authors (MH, NT, and ST) to allow immersion in the data. A thematic framework was developed by reviewing the notes developed during the familiarization process and identifying key themes and subthemes to create a hierarchical framework. The framework was then systematically applied to all the data (known as indexing), concurrently modifying and refining the framework to maximize the grounding of the framework in the data. A series of thematic charts were subsequently devised with indexed data entered into an appropriate cell (charting) to establish the interconnectedness of the themes. This stage assisted the mapping and interpretation, which involved identification of patterns and key issues by making comparisons and developing explanations that were grounded in the data. An example of the thematic charts is shown in Table 1.

**Table 1:** **Example of the development of the core concept of ‘practical problems’**

**Codes**	**Themes**	**Core concept**
Positioning mirror	Positioning self and mirror	Practical problems
Positioning self		
Distracting environment	Environmental difficulties	
General ward clutter		
Not enough time – patients	Lack of time	
Not enough time – therapists		
Not enough time – nurses		
Lack of therapy support from nursing staff	Involve others	

## Results

Five focus groups were undertaken at five locations attended by 3 to 13 participants with more than one stroke service represented at each group. All the recruiting sites were represented. Participants included physiotherapists (n = 15), occupational therapists (n = 5), nurses (n = 8), and doctors (n = 2). Staff grades ranged from a student therapist and junior staff (Band 5) to senior specialist therapists and nurses (Band 8) plus two senior (consultant) stroke physicians. Details of the participants are shown in Table 2.

**Table 2:** **Details of the participants in the focus groups**

	***Seniority***	***Sex***	***Home site***
**Focus Group 1**			
Physiotherapist	Band 6	Female	Site 10
Physiotherapist	Band 5	Female	Site 10
Occupational therapist	Band 7	Female	Site 10
Physiotherapist	Band 7	Female	Site 10
Research nurse	Band 6	Female	Site 1
Research occupational therapist	Band 6	Female	Site 4
Senior research nurse	Band 7	Male	All Sites
Stroke care coordinator	Band 6	Female	Site 11
Senior research nurse	Band 6	Female	Sites 4, 5 & 7
Research nurse	Band 6	Female	Sites 9 & 6
**Focus group 2**			
Research nurse	Band 6	Female	Site 3
Physiotherapist	Band 8	Female	Site 3
Physiotherapist	Band 8	Female	Site 3
Research nurse	Band 6	Male	Site 2
Physiotherapist	Band 7	Female	Site 2
Physiotherapist	Band 8	Female	Site 2
**Focus Group 3**			
Research physiotherapist	Band 6	Female	Site 1
Research nurse	Band 6	Female	Site 1
Physiotherapist	Band 7	Female	Site 1
Consultant stroke physician	-	Female	Site 1
**Focus Group 4**			
Consultant stroke physician	-	Male	Site 4
Physiotherapist	Band 7	Male	Site 7
Occupational therapist	Band 7	Female	Site 4
Physiotherapist	Band 5	Male	Site 5
**Focus Group 5**			
Physiotherapist	Band 7	Female	Site 11
Occupational therapist	Band 7	Male	Site 11
Physiotherapist student	Final Year	Female	Site 11
Physiotherapist	Band 5	Female	Site 12
Occupational therapist	Band 6	Female	Site 12
Physiotherapist	Band 7	Female	Site 8

Band 5, junior grade; Band 6, senior grade; Band 7, specialist grade; Band 8, head/senior specialist grade.

Five themes emerged from the analysis: the effect of the interventions; practical problems; patient-related factors; professional dilemmas; and skills. Possible solutions were identified for each barrier to implementing the patient-led interventions. Each theme is discussed in more detail below.

### The effect of the interventions

When developing the randomized controlled trial of patient-led therapy, the effects of the interventions were anticipated to be on the patients’ motor impairments and activity limitations, and outcome measures were chosen to address these. However, while recognizing the difficulty of detangling the effect of the new interventions from those of ‘traditional’ therapy, staff felt patient-led therapy focused more frequently on patients’ psychological well-being. They identified a beneficial effect on the patients’ sense of autonomy, explaining that it enabled patients’ to take some control over their situation and their therapy. It also helped to keep patients occupied as they were alone and inactive during much of the day. For many patients, this was reported to have a motivating effect that was enhanced by signs of recovery:

*“We’ve had a lot of patients who have had no movement at all …….and even if they had a flicker, it’s a great achievement for that person and it’s a great motivation.*” Focus Group 1: Participant 5

Several other therapists felt the main effect was on spatial inattention or neglect (a cognitive impairment) rather than motor impairments.

### Practical problems

Staff identified several practical problems with delivering patient-led therapies. Patients were reported to often struggle to get themselves and the equipment in to the correct position, particularly those with more severe impairments and those doing mirror therapy. In addition, the bedside tables were too small to position the mirror and/or exercise booklet so the patient could see their arm (mirror therapy), and it was difficult to make space in the general clutter of the bedside (magazines, water jugs, spectacles, *etcetera*). A solution at some sites was to set up the equipment for mirror therapy on a large table (such as a dining table) in a quiet room, which would allow patients to more easily get in the correct position and to concentrate. This provided a less distracting environment for those with concentration difficulties and worked well for those who were mobile, but was less successful for those who needed assistance to get to the room.

A stroke unit is a busy environment, and staff perceived they sometimes did not have time (or could not give priority) to the light touch supervision. Some therapists overcame this, to some extent, by setting the patients up for the therapy at the end of their usual therapy sessions either in the therapy room or at the bedside.

All participants found that involving others was key to supporting patients to complete the therapy. This included relatives or visitors who supervised the patients during their visits, student nurses and therapists, and all members of the multidisciplinary team. Perceptions of the feasibility of involving other members of the team appeared to depend on the strength of the existing team. Where interprofessional relations were positive, this was viewed as a solution to facilitate the therapy and enhanced team working. In contrast, where working relationships were less well-developed, involving other members of the team was viewed as a barrier preventing uptake. Involving others was thought to work most effectively when therapy or rehabilitation assistants were tasked to take responsibility for the supervision. They became the main contact point for patients and staff regarding the patient-led therapy and gave it priority in their workload.

Establishing a routine so that patient-led therapy became normal practice for staff and patients was also important. When available, patient timetables were helpful as these enabled staff and patients to identify a specific time each day when the patient should complete the therapy, and this time was protected from other demands:

*“*[We] *reinforced to the patients, really instilling what they should be doing and getting into a routine of doing it”* Focus Group 5: participant 3

*“a lot of patients have reported that “oh I do my exercises at this time”, and you’ll see them doing it at the same time every day”* Focus Group 1: participant 3

### Patient-related factors

Although staff were generally positive about the notion of patient-led therapy, they recognized that it was difficult, and sometimes not feasible, for some patients. Those with cognitive impairments (such as memory, concentration, problem-solving and initiation difficulties and neglect) often found it difficult to complete to complete the therapy. Moods were also a problem; those with severe depression and/or who were emotionally devastated by their sudden illness were perceived to be unable to initiate patient-led therapy. Severe weakness, limited sitting balance and visual deficits (such as hemianopia) made the patient-led therapy physically challenging. For others, the difficulty lay with patients’ motivation and self-efficacy. There was a need to manage patients’ expectations, as staff felt many lacked motivation and had an external locus of control:

*“I think it’s a complete change from what patients think about therapy, a lot of them* [the patients] *seem to think that it should be done to them, as opposed to something they initiate.”* Focus Group 1 Participant 5

More selective inclusion criteria were considered the main solution. There was also a need to develop strategies to promote the self-efficacy to undertake independent exercise, although staff were unsure how to go about this. Again, involving others to prompt and reiterate the importance of the patient-led therapy was felt to be important, as was establishing a routine.

### Professional dilemmas

Encouraging patients to exercise in their own time and work independently contrasted strongly with the usual way therapy was delivered, in which a therapist works with the patient in a highly controlled environment using their expertise to assist (or facilitate) the patient to move or work in a very specific way [19,20]. It was considered detrimental to patients’ overall progress to allow them to move in a different way. Not surprisingly, many therapists found this change a challenge. Concern was expressed about relinquishing control of the patients’ activity, particularly that patients may do the exercises incorrectly or may overdo it, as undertaking effortful activities was considered detrimental by some staff.

*“It didn’t, necessarily, tie in with how we handle patients. You know, it’s completely alien… They* [the patients] *would always need facilitating and handling”.* Focus Group 2 Participant 2

Staff dealt with this reluctance to relinquish control in different ways. Some stated that, as the interventions were patient-led, they left the therapy up to the patients entirely and did not provide light touch supervision. They then continued to provide their therapy in one-to-one controlled sessions, as usual. This meant that changes to their usual practice were minimal. Others felt they could not leave the patients to work independently (particularly those with the challenges identified in the ‘patient related factors’ above). They gave more than light touch supervision during the ‘patient-led therapy’ by providing hands-on assistance (or facilitation) during the exercises so they were performed in the manner the therapists found acceptable. This was time consuming and was sometimes performed instead, rather than in addition, to their usual one-to-one therapy sessions.

Difficulty juggling the patient-led therapy with other professional priorities was also raised. The new intervention took some time and effort to supervise and this was felt to detract from delivering ‘real’ standard therapy or other clinical demands and priorities. If the patient-led therapy was added, the therapists needed to decide what to take out of their usual care to make time.

*“If we’re spending a lot of our time trying to set* [the patient] *up and encourage* [them] *with that, it’s going to impact on how much other therapy we’re doing”* Focus Group 1, Participant 6

*“Yes, when do we decide that the mirror therapy becomes more important than the rest?”* Focus Group 1 Participant 7

There was also some conflict with other policies. Therapists were under pressure to increase the amount of ‘traditional’ face-to-face therapy delivered and making time to supervise the patient-led therapy detracted from this. The most common solution was to involve nonqualified therapists (assistants), other members of the team, visitors or hospital volunteers. Some were concerned with whether the patient-led therapy would impact on other hospital policies such as infection control, health and safety and manual handling/ falls prevention. Others were concerned that the patient-led therapy may have a detrimental effect on mood and motivation. Although distressing this could also be part of the grieving process of coming to terms with limited recovery.

*I actually found it* [patient-led therapy] *had a negative impact on this patient, because he had so little function on his affected side, it was just sheer frustration. It was almost as though it was highlighting to him his lack of the mobility, and I think it really upset… So the ones with very limited movement it’s going to be quite a soul destroying existence.”* Focus Group 5 Participant 4

*“I'm always wary of giving people false hope, if somebody with a really dense arm weakness, they might latch onto this and think “that's going to make my arm better”..... Then it worries me, if their arm doesn't get better, it'll knock them off. But in fact it takes their mind off it, instead of sitting there with their arm not doing anything and then thinking about it. Actually, they've got something to do, it takes their mind of it, and even if it doesn't come back I suppose it'll help with the grieving process a bit of losing the functional element.”* Focus Group 4 Participant 1

### Skills

Connected to the professional dilemmas of introducing a new type of intervention in to their practice, staff expressed a lack of confidence to do so. Although they had all receiving the same training package, which was more detailed and sustained than many continued professional development opportunities in clinical practice, some felt ill-equipped to deliver the treatments. They particularly wanted more details about how to deliver the interventions and how to adapt them to individuals or different contexts.

*“We really didn’t know how much prompting we should be doing or guidance we should be giving with it being a self-initiating programme. I would certainly say that that was something I wasn’t confident in”* Participant 6 Focus Group 3

*“After I had that reassurance off her* [the research therapist] *……that was okay. I could sort of judge it for myself a little bit more”* Participant 5 Focus Group 3

*“It doesn’t have to be set “this is what we’re doing” for the whole research, you can change it and see how it works”* Participant 5 Focus Group 3

## Discussion

The findings of this study illustrate the complexity of providing a new intervention during a randomized controlled rehabilitation trial and offer some solutions to barriers. Participants highlighted that the perceived effects of the intervention, practical problems, patient-related factors, professional dilemmas, and skills affected whether, or how, the trial intervention (patient-led therapy) was delivered. They found that it was helpful to establish a routine that embedded the intervention into everyday practice, involve others and build relationships with the whole multidisciplinary team, carefully select criteria and adapt the environment to facilitate implementation. They also highlighted the challenges of juggling professional and other clinical priorities with the drive to deliver the trial, the uncertainty and perceived lack of skill about how to deliver the intervention, the need to adapt to clinical needs and priorities, and the need to change practice to overcome barriers.

The main findings are congruent with our report of patients’ experience of patient-led therapy during inpatient stroke rehabilitation (unpublished data) and previous reports of fidelity in stroke rehabilitation trials [21]. The most similar previous report [22] focused on developing a theoretical framework of fidelity (based on the Consolidated Framework for Implementation Research) as part of a randomized controlled trial of occupational therapy for people with stroke living in care homes. In both trials, staff groups highlighted similar challenges to meet the demands of balancing their professional responsibilities with delivering the trial protocol. They also concurred on factors promoting fidelity, engaging the whole care team, building relationships, embedding the trial interventions in to routine care, and reorganizing the environment. It is noteworthy that these factors are also highlighted in the literature on implementing change (also referred to as service improvement or development) in clinical practice, whether taking a theoretical approach (for example, the Normalization Process Theory [23]) or a more pragmatic approach, as illustrated in recent National Institute of Clinical Excellence Guidance [24].

The occupational therapists in the care home trial did not describe the lack of confidence to deliver the interventions in the same way as our focus group participants. This may be because they were delivering familiar interventions in a new environment, whereas our participants were delivering a new intervention. They did however highlight how their skills and confidence improved over time. This ‘learning curve’ has been noted previously, primarily in trials of surgery [25,26] and when implementing new interventions into clinical practice [23], and may also be an important factor in trials of rehabilitation and other complex interventions.

Patient selection was less of an issue for the occupational therapists in the Occupational Therapy in the care home trial, possibly because it was a cluster trial, so all patients were included. As MAESTRO was a feasibility trial, we made the inclusion criteria as broad as possible to explore which patients could, or could not, adhere to the interventions. Consequently, staff reported many patient-related factors that limited application. In future trials and clinical practice, we would exclude patients whose cognitive, mood, motor or balance impairments precluded participation. We would include elements to promote and support self-efficacy and autonomy while teaching patients how to undertake the therapy. This also needs to be explicitly addressed in the training package for staff, with support and strategies to manage patients’ expectations (both positive and negative) [27,28]. It is noteworthy that the focus group participants viewed the main impact of the patient-led therapy to be on patients’ motivation and autonomy (a view that was shared by the trial patients in interviews of their experience), rather than on the impairments and activity limitations that were the outcomes measured in the trial. This has important implications for treatment fidelity as if staff and/or patients feel the intervention is serving a different purpose to that in the trial proposal, they are unlikely to deliver it in the way intended. It also highlights the importance of fully piloting interventions and obtaining users (professionals and patients) feedback before a definitive trial. In future trials of patient-led therapy, we would include self-efficacy and mood as outcomes.

When developing the staff training package, we aimed to be informal and flexible, believing this would make the training acceptable and accessible. However some staff seemed to fail to recognize that they had received training and felt their preparation to deliver the interventions and deal with difficulties was incomplete. A more formal training package, possibly with time away from the clinical environment, certificates of attendance, and ongoing support to overcome difficulties as they occurred may have more effectively enabled staff to build their confidence and skills to deliver the interventions. Objective monitoring and feedback on competence would promote fidelity to the research protocol, but is alien to current practice and would be off-putting for many staff. Relatively high staff turnover as junior staff rotate or people move posts also means the training package needs to be available in the long term (possibly using electronic learning methods). Staff’s need for on-going support and training further illustrates the need to accommodate the learning curve to deliver a new skill in practice.

The demand to deliver the patient-led therapy also needed to be juggled with the demand to deliver other clinical policies and targets. Suggested solutions were to involve therapy assistants, other members of the multi-disciplinary team and visitors in supervising the patient-led therapy. For some participants, this was considered a positive development, which enhanced productive working relationships across the multidisciplinary team, but others, possibly where existing inter-professional relationships were not strong, viewed it as a major barrier. Future trials need to consider the impact of patient-led therapy on the multidisciplinary team and other aspects of stroke rehabilitation, and the training package needs to promote strategies to enhance and enable this.

Patient-led therapy is an innovation that contrasts with traditional ways of delivering in-patient rehabilitation, and staff needed time and support to build the necessary skills and strategies, adjust attitudes and develop confidence. This had an impact on staff’s clinical judgement. New ways of working that relinquished control over patients’ activity were required. This could diminish therapists’ status within the multidisciplinary team and with patients, and many were uncomfortable with it. This is a common issue among healthcare professionals when faced with interventions to promote patients’ self-management [29,30]. Staff needed to make professional decisions about which aspects of their usual practice to discard to make way for the new interventions, and judge how to prioritize the new interventions (with unknown efficacy) against their usual ways of working, which they considered efficacious. Future trials of patient-led therapy need to include a generous period for staff to overcome this learning curve and support to change their way of thinking [29,30].

These professional dilemmas need to be acknowledged and given time and support to resolve them. Yet the challenge of implementing changes in practice, and the resources needed to support them are rarely acknowledged in trial protocols or in the literature regarding fidelity [31-33]. This is an area where trial methodologies can learn from implementation science where its importance is widely acknowledged [23]. The notion of a research protocol that specifies exactly how an intervention should be delivered contrasts with the reality of delivering complex interventions in the ‘real world’ where adaptability to individual needs and the local context is considered a fundamental aspect of clinical expertise. This may require a change in approach to protocol fidelity. A more acceptable approach may be to define the essential aims, objectives and elements of the interventions(s) and then work iteratively with clinical staff to identify strategies,, overcome boundaries to flexibility, and build confidence.

The main limitation of this study is the generalizability of the findings; one cannot assume the same issues will emerge with other research teams, individuals, interventions or clinical services. However we have included members of the multidisciplinary teams from inpatient stroke services (with varied seniority, experience, post-graduate education and length of service) across a conurbation serving more than 3 million people who use varied service models, so we cautiously feel the results are reasonably representative of the staff who may be called on to deliver patient-led interventions for in patients with stroke. Further work is needed to consider whether the main points raised here generalize to other settings and clinical conditions.

## Conclusions

Delivering complex interventions within a trial is complex. Staff require time and support to develop the skills, strategies and confidence to identify suitable patients, deliver new treatments, adapt the new treatments to individuals’ needs, and balance the demands of delivering the trial intervention according to the treatment protocol with other clinical and professional priorities.

## Abbreviation

MAESTRO: Mirror arm exercises after stroke trial
